# Emotional Intelligence and School Bullying Victimization in Children and Youth Students: A Meta-Analysis

**DOI:** 10.3390/ijerph20064746

**Published:** 2023-03-08

**Authors:** Yijing Zhang, Ji-Kang Chen

**Affiliations:** Department of Social Work, The Chinese University of Hong Kong, Hong Kong

**Keywords:** bullying, victimization, emotional intelligence, children, youth students, meta-analysis

## Abstract

School victimization among children and youth is a global public health issue that has long-term adverse effects on the victims’ mental health and behavioral development. Theories and research suggest that emotional intelligence may operate as a buffer against school bullying victimization. However, the strength of the association between emotional intelligence and bullying victimization is controversial. Therefore, we aimed to conduct a meta-analysis to evaluate the exact association between Emotional intelligence and school bullying victimization. We conducted a systematic search in PubMed, Web of Science, ProQuest Dissertations, Google Scholar, and China National Knowledge Infrastructure (CNKI) from inception to March 2022 for relevant studies that examine the association between emotional intelligence and school bullying victimization without a language limit. Twenty-four articles were included in our meta-analysis (*n* = 27,438). There was a small, negative, and significant association between emotional intelligence and school victimization among children and youth students. Sex and emotional intelligence measurement tools were variables that significantly moderated the link between emotional intelligence and bullying victimization. The findings indicated that improving students’ emotional intelligence could be a crucial strategy to lower the students’ risk of being bullied in school and online. It would be more effective among male students.

## 1. Introduction

School bullying victimization among children and youth is a global public health issue that negatively affects student the victims’ mental health and behavioral development [1,2,3,4,5,6,7]. Common forms of school bullying victimization discussed in the literature include physical (e.g., being hit and kicked), verbal (e.g., being name-called, cursed, and insulted), relational (e.g., having rumors spread about them and being socially excluded), sexual (e.g., being sexually harassed), and cyber types (e.g., bullying through electronic communication tools). Physical, relational, verbal, and sexual bullying are frequently referred to as “traditional” forms of bullying [1,8]. Thus, bullying is categorized into two types in this study: traditional bullying and cyberbullying, in this study. School bullying victimization is reported by between 15% and 23% of elementary students and between 20% and 28% of secondary school students in the USA [9,10,11].

An increasing number of interventions have been developed recently to reduce bullying and victimization in educational settings [12,13,14]. However, little is known about the factors that are required to improve the effectiveness of interventions for reducing school bullying victimization. Theories, such as emotional theories [15,16,17,18] and the trait emotional intelligence theory [19], have argued that emotional intelligence is one of the major protective factors against school bullying victimization. Thus, emotional intelligence is a variable that may be significant to consider when one is designing these interventions. Recently, there was a systematic review and meta-analysis that examined the association between emotional intelligence and adolescents’ aggressive behaviors [20]. However, no meta-analysis has systematically examined the strength of the association between emotional intelligence and bullying victimization in the children and youth student population, making it difficult to form a broader picture of what real benefits can be derived from introducing intervention programs that promote emotional abilities to buffer adolescents against bullying victimization.

Furthermore, previous studies have examined the association between emotional intelligence and bullying victimization. Mavroveli and colleagues [21] found a positive link (r = 0.054) between emotional intelligence and bullying victimization among elementary school boys in the UK. Peachey and colleagues [22] found a positive correlation between emotional intelligence and bullying victimization among elementary school girls in the USA. However, Hsieh and colleagues [23] investigated 6233 elementary school students and found a negative link between emotional intelligence and bullying victimization (r = −0.12). Similarly, Kokkinos and Kipritsi [24] also found a negative correlation between emotional intelligence and bullying victimization among elementary school children in Greece (r = −0.260). Thus, the link between emotional intelligence and bullying victimization is not conclusive.

### 1.1. The Association between Emotional Intelligence and Bullying Victimization

Emotional intelligence (EI) indicates the subset of social intelligence that involves several emotion-related abilities: the appraisal and expression of emotions in self and others, regulation of emotions in self and others, and utilization of emotions in problem solving [25]. EI has been considered as an influential factor in predicting bullying victimization [26,27,28,29,30]. People with high EI are more likely to deal with negative events appropriately than people with low EI are [26]. Several emotion theories suggest that emotions have a profound influence on perception, cognition, and action, and emotions also serve adaptive purposes [15,16,17]. These emotion theories [15,16,17,18] and Trait EI theory [19] all support trait emotional self-efficacy that motivates one to adapt coping behaviors at school. High trait EI scores may aid in adaptive coping with social interactions and may protect against the development of maladaptive behaviors, such as bullying [31]. Furthermore, teenagers with high EI have better social relationships [32], and stronger peer competence [33] than those with low EI do. Many studies have examined the link between EI and school victimization and consistently found a negative correlation between these two variables among secondary school students [34,35].

However, some other researchers have argued that the association between EI and bullying victimization is not negative, but positive. Victims with high EI may be more emotionally aware, but less capable of perceiving the emotions of their peers [36]; therefore, they may be disliked by peers and more likely to be bullied [37]. For instance, cross-sectional studies have revealed a positive association between emotional intelligence and peer-reported school bullying victimization of elementary school boys in the UK [21]. Therefore, the nature of the link between EI and bullying victimization remains ambiguous.

### 1.2. Impact of Moderator Variables

In the literature, emotional intelligence has been assessed using various measurement tools. Among them, the three models proposed by Joseph and Newman [38], namely performance-based ability, self-reported ability, and self-reported mixed models, are the most common theoretical frameworks used to discuss differences in assessing EI measurement tools. The performance-based ability model views EI as an emotional aptitude, a mental ability that combines reasoning about our emotions focused primarily on processing hot (emotionally laden) information [39], measured by the “Mayer-Salovey-Caruso Emotional Intelligence Test” [40]. The self-reported ability model, which considers EI to be a mixture of emotional aptitudes, employs self-reported instruments asking the participants to rate their own EI subjectively [41]. The main scales are TMMS [42], SUEIT (Swinburne University Emotional Intelligence Test) [43], and WLEIS (Wong Law Emotional Intelligence Scale) [44]. WLEIS is the most utilized scale. The self-reported mixed model views emotional intelligence as encompassing (among others) goals, interpersonal talents, empathy, personality traits, and well-being measured by EIS (Emotional Intelligence Scale [45]) and Bar-On Emotional Quotient Inventory [46]. Even though alternative measures exist, researchers primarily employ TMMS, WLEIS, EIS, and EQI to examine the association between emotional intelligence and school bullying victimization. Because the variations in the scales are likely to influence the results, this study investigated the moderating effect of emotional intelligence measurement tools on the link between emotional intelligence and bullying victimization.

The association between EI and school bullying victimization may differ according to bullying victimization types. According to trait EI theory and emotion theories, high trait EI scores may aid in adaptive coping with social interactions and may protect against the development of maladaptive behaviors, such as bullying [15,16,17,18,19]. Since cyberbullying is considered to be a type of bullying, the link between EI and cyberbullying victimization should be negative. However, in the context of the virtual world, where cyber users, including bullies and victims, are unable to interact physically with other participants [43], cyberbullying victims may not be affected by emotional intelligence. Many studies have tested the association of EI with traditional bullying and cyberbullying victimization. For example, Rey et al. [47] revealed that higher levels of total EI were negatively and significantly related to lower student cyberbullying victimization rates. However, EI and cyberbullying positions, including victims, do not seem related in an empirical study conducted in Tehran [48]. Nevertheless, Alvarado et al. [49] found that kids with high EI scored the lowest on a traditional bullying victimization scale. Therefore, how school bullying victimization types affect the association between emotional intelligence and bullying victimization is unknown.

Traditional bullying victimization is frequently measured using two scales. First, the peer relation questionnaire (PRQ) was created specifically for children and adolescents to assess bullying tendencies and the incidence of being victims who are targeted by peers [50]. Second, the Peer Victimization Scale (PVS [51]) is a 16-item self-report scale assessing physical victimization, social manipulation, verbal victimization, and damage to property as classic bullying victimization behaviors. The European Cyberbullying Intervention Project Questionnaire (ECIPQ [52]) is the most widely used measurement tool for determining whether a person has been a cyber victim of specific online behaviors in the previous two months. Most studies evaluating the association between emotional intelligence and bullying victimization have used PRQ, PVS, and ECIPQ, despite the availability of other measures. The above scales have different foci and produce varied outcomes due to their different theoretical bases. The present study examined how bullying victimization measurement tools influence the association between emotional intelligence and bullying victimization.

The association between EI and school bullying victimization may differ by age. For example, the group socialization development hypothesis [53] argues that as students mature, they become more concerned about the opinions of others and come to realize that bullying is unacceptable. Therefore, the association between emotional intelligence and bullying victimization may decrease with age/grade level. However, according to social learning theory [54], as children become older, they are associated with more peers who engage in risky behaviors, thus, they may mimic those behaviors and are more likely to engage in school bullying. Martínez-Martínez et al. [55] indicated that the correlation between EI and bullying victimization was large and the highest among senior students (r = −0.45). However, Elipe and colleagues found a moderate link between EI and bullying victimization among senior students (r = −0.21) [28]. Until now, the link between emotional intelligence and bullying victimization appears to show different trends with age, and no concrete conclusions can be drawn. Therefore, through a meta-analysis, this study further examines how age/grade level affects the link between emotional intelligence and bullying victimization.

Previous studies have also shown that the association between emotional intelligence and school bullying victimization may differ by culture. For example, some studies have revealed a stronger link between EI and bullying victimization among Asian students than they have among American or European students [22,23,56]. However, some research has found a poorer association between emotional intelligence and bullying victimization in Asian and European/American pupils than it has in Australian students [35,57,58,59]. This study examined whether culture affects the link between emotional intelligence and bullying victimization through a meta-analysis.

In addition to culture, sex might also be a potential moderating factor. Males and females may differ in emotional intelligence based on their educational experiences and socialization processes throughout childhood [60,61]. Females exhibit EI at higher levels than males do [62,63]. Moreover, the prevalence of bullying victimization is lower among females than it is among males [64,65,66]. Thus, the association between EI and bullying victimization may differ across sex. Inconsistent results have also been found regarding the effect of sex on the association between emotional intelligence and school bullying victimization. For example, Mavroveli and colleagues found that the link between EI and bullying victimization was positive for males and negative for females when they were tested using a peer assessment scale [21]. However, Peachey and colleagues found the link was negative for males and positive for females [22]. Therefore, one aim of this meta-analysis is to reconcile these inconsistent findings.

### 1.3. The Current Study

The purpose of this study was to examine the strength of the association between EI and bullying victimization. This study investigated relevant cross-sectional studies such as various EI models [38] and different forms of bullying victimization studies [67]. In addition, past studies have shown that differences in the strength of the association between emotional intelligence research and school bullying victimization may be influenced by the measurement tools or participant demographic characteristics [68]. However, most previous studies have rarely considered how measurement instruments or demographic factors moderate the association between EI and bullying victimization. Therefore, this study examined whether the link between emotional intelligence and bullying victimization varied by the (a) EI measurement tools, (b) bullying victimization measurement tools, (c) bullying victimization types, and (d) demographic characteristics of the sample (sex, culture, and grade level). In this meta-analysis review, we did not select for other potential moderators (i.e., parenting style, race/ethnicity) because these variables are rarely reported in the related literature. To our knowledge, this study would be the first meta-analysis to examine the association between EI and school victimization among children and youth students.

## 2. Methods

We followed the Meta-Analyses (PRISMA) statement [69] when we were conducting this meta-analysis. We retrieved all cross-sectional studies that assessed the associations between emotional intelligence and bullying victimization before conducting this quantitative synthesis without a language limitation.

### 2.1. Literature Search

We extensively searched electronic databases from inception to March 2022 for related documents to find research on emotional intelligence and bullying victimization, including PubMed, Web of Science, ProQuest Dissertations, Google Scholar, and China National Knowledge Infrastructure (CNKI). The following search terms and search algorithms were used: (Emotional intelligence OR Emotional competence) AND (bullying victimization OR peer victimization OR cyber victimization OR cyberbullying victimization OR bullying OR victimization OR cyberbullying OR bullies OR victims OR bully-victims OR cyberbullies OR cybervictims) NOT workplace. There was no restriction on publication language or region.

### 2.2. Inclusion Criteria and Exclusion Criteria

To ensure the study’s accuracy, only articles that met the following five criteria were included: (a) the research was a quantitative study; (b) emotional intelligence was an independent variable; (c) traditional bullying victimization and/or cyberbullying victimization were the outcome variables; (d) the research targets were primary school, secondary school, or university school students; (e) the researchers reported an r between emotional intelligence and bullying victimization (or an F that can be transformed into r).

The exclusion criteria listed below were used: (a) wrong studies’ subjects that are unrelated to school bullying victimization (including cyberbullying victimization), such as domestic violence victimization, sport teammate bullying victimization, and abuse victimization; (b) wrong studies’ samples outside of elementary school students, secondary school students, and university students; (c) wrong article types, such as conference papers, reviews, protocols, etc.; (d) study methodologies that are not cross-sectional (e.g., a randomized control trial study); (e) studies that focus on social-emotional intelligence or social emotional competence instead of emotional intelligence; (f) studies that only measure a sub-dimension of emotional intelligence; (g) the full-text was not available; (h) studies that use the same sample; (i) there are not sufficient data to calculate the effect size between emotional intelligence and bullying victimization.

### 2.3. Article Selection

Initially, two researchers independently searched for the articles and selected those that met the inclusion criteria by screening the titles and abstracts. Any discrepancies in study eligibility were solved through conversation. The title and abstract of studies were first parts used to determine their eligibility. After that, the complete text was used to screen all the appropriate studies.

### 2.4. Data Extraction and Management

The information from the chosen papers was independently extracted by two researchers. A standardized form including the following information was used to organize all the chosen data: (i) the sample size; (ii) the first author’s last name and the year of publication; (iii) mean age; (iv) female percentage, if available; (v) culture; (vi) grade level; (vii) school bullying victimization measurement tools; (viii) emotional intelligence measurement tools; (ix) school bullying victimization types; (x) r.

We coded the included studies according to the following criteria: (a) the effect sizes were coded for every independent sample; (b) if a study reported not only different dimensions of emotional intelligence and bullying victimization, but also a total of emotional intelligence and bullying victimization, we only coded the latter one; (c) if a study gave several effect sizes for sample characteristics, such as sex, we coded the mean value of the effect sizes; (d) grade level were divided into elementary school students, secondary school students, and university students; (e) if a study used several scales to measure bullying victimization, we only chose the r related to the most commonly used bullying victimization scales; (f) if a study reported both r of EI-cyberbullying victimization and EI- traditional bullying victimization, we coded the mean value of the effect sizes; (g) when several studies used different years’ versions of the same scale, we categorized these scales together; (h) when a study reported the correlation coefficient between EI and bullying victimization in the total sample (male cohort and female cohort separately), we used the total value instead of the others.

### 2.5. Quality Assessment

We used the JBI Critical Appraisal Checklist for Analytical Cross-Sectional Studies to assess the studies’ quality [70]. The checklist has 8 items, and the answers “Yes”, “No” “Unclear” or “Not applicable” are given to each item. The total number of “yes” responses was added, multiplied by 100, and divided by the total number of items for each study. Each study’s total item count was adjusted by deducting “N/A” responses.

### 2.6. Statistical Analysis

Comprehensive Meta-Analysis 2.0 was used for the meta-analysis. The homogeneity test and mean effect were estimated using a random effects model. Mean effect sizes were calculated using the averaged correlation coefficients of independent samples. The effect size of each study and the overall meta-analysis were displayed using a forest plot. To determine whether there were differences between the included studies, we tested the heterogeneity using a Q statistic with *p* < 0.1 indicating heterogeneity between the studies, and the *I*^2^ test, which explains the percentage of variability among the effect sizes beyond that which is expected by chance.

When homogeneity test revealed a significant difference, moderators were tested: EI measurement tools, bullying victimization measurement tools, bullying victimization types, culture, grade level, and sex. Sub-group analyses were conducted to test the categorical moderators, while meta-regression was conducted to test the continuous moderators. We followed the guidelines regarding the minimum number of studies needed for moderator analysis to reduce the risk of false-positive effects. To assess the presence of a moderator variable, there must be at least three required entries in order to consider variability for the chi-square analysis [71]. We only performed the meta-regression when there were more than ten studies (k) [72]. Publication bias refers to the possibility that studies with insignificant results are less likely to be published. We use a funnel plot, Rosenberg’s Fail-Safe analysis, and Egger’s test to examine whether there was a publication bias.

## 3. Results

### 3.1. Descriptive Results

**Figure 1:**
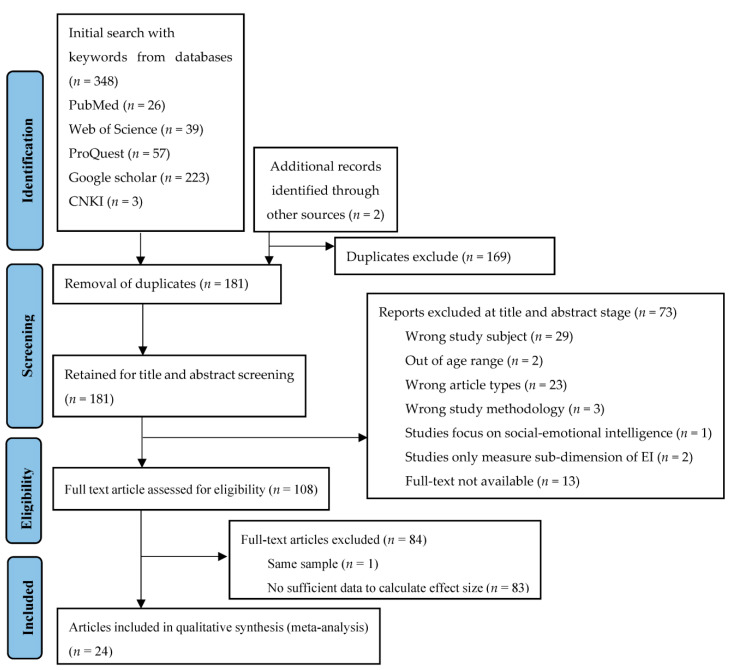
PRISMA flow chart of study selection.

Our review of the literature yielded 350 papers. After that, 169 duplicates were removed. The remaining 181 literature were reviewed for title and abstract, with 73 being rejected due to the exclusion criteria. One hundred and eight studies were submitted for full-text screening, with eighty-four of them being excluded. The process is shown in Figure 1.

### 3.2. Characteristics of the Included Studies

This meta-analysis of 24 articles had 27,438 participants (Table 1). The range of the sample sizes was from 68 to 6233. The mean age of study participants ranged from 9.30 to 20.45 years, and the female percentage ranged from 21% to 68%. The studies were conducted in two different cultures, with the most of them taking place in Western countries (*n* = 20), and the others in Eastern countries (*n* = 4). The grade level categories of the participants included in the study were elementary school students, secondary school students, and university students. There are two types of bullying victimization: traditional bullying victimization and cyberbullying victimization.

### 3.3. Effect Size and Moderator Analysis

**Figure 2:**
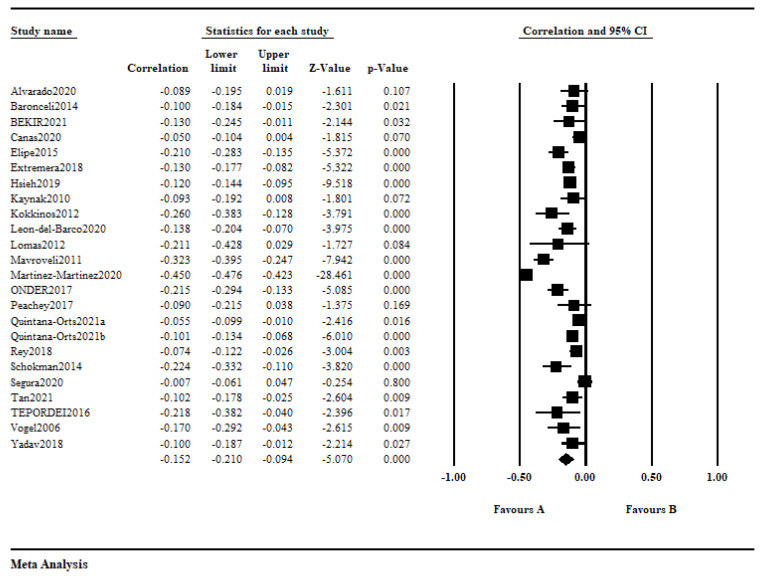
Forest plot of the association between emotional intelligence and bullying victimization [21,22,23,24,26,28,29,34,35,47,49,55,56,57,58,59,73,74,75,76,77,78,79,80].

The inconsistency of the homogeneity test revealed significant heterogeneity among the examined studies (*Q* =502.541; *p* < 0.001; *I*^2^ = 95.423). The random-effect model showed an overall pooled effect size of −0.152 (95% CI: from −0.210 to −0.094) between emotional intelligence and bullying victimization. According to the effect size criteria from Rice and Harris [81], −0.152 is a small and negative effect size. As can be seen in Figure 2, the r is range from −0.007 to −0.450.

A variation meta-analysis was performed to determine if the EI tools, bullying victimization tools, bullying victimization types, culture, and grade level affected the link between emotional intelligence and bullying victimization. Emotional intelligence measurement tools moderated the association between EI and bullying victimization significantly (EIS, EQI, TMMS, WLEIS, and SUEIT; *Q* = 25.691, *df* = 4, *p* < 0.001). The bullying victimization measurement tools did not significantly moderate the association between EI and bullying victimization (ECIPQ, PRQ, and PVS; *Q* = 6.739, *df* = 2, *p* > 0.05). The types of bullying victimization had no significant effect on the association between emotional intelligence and bullying victimization (*Q* = 0.062, *df* = 1, *p* > 0.05). Grade level did not influence the association between emotional intelligence and bullying victimization (*Q* = 0.645, *df* = 1, *p* > 0.05). Bullying victimization was not significantly associated with emotional intelligence across cultures (Western countries and Eastern countries) (*Q* = 1.238, *df* = 1, *p* > 0.05). These findings are presented in Table 2.

The meta-regression analysis revealed that sex significantly moderates the connection between emotional intelligence and bullying victimization (*Q_Model_* (1, k = 24) = 502.541, *p* < 0.001) (Table 3), which shows the strength of r decreases as the percentage of females increases.

### 3.4. Publication Bias

**Figure 3:**
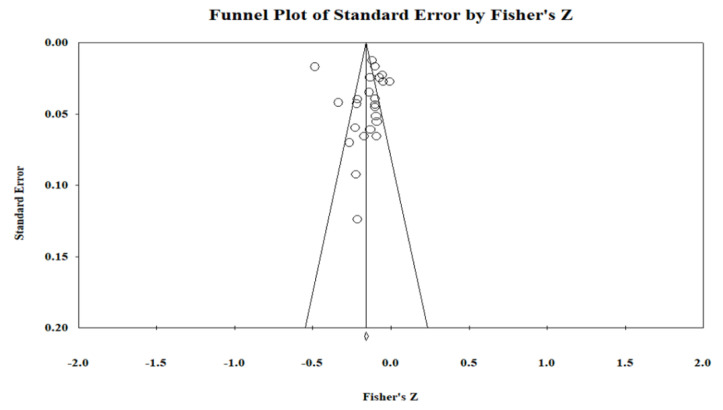
Result of funnel plot publication bias analysis.

As displayed in the funnel plot (Figure 3), the data are symmetrically distributed around the overall effect size, and Egger’s test showed that publication bias is improbable (*p* = 0.774 > 0.05). Rosenberg’s Fail-Safe N analysis revealed that to lower the *p* value to a non-significant level (>0.05), 2989 missing studies with effect sizes of zero were needed. As a result, no evidence of publication bias was discovered using these approaches.

## 4. Discussion

Overall, emotional intelligence and school bullying victimization were found to have a small, negative, and significant relationship (r = −0.152, *p* < 0.001) among children and youth students. Participants with lower EI were more likely to be bullied. This finding aligns with theories and research suggesting that emotional intelligence protects against bullying victimization in schools and that people with high EI are more likely to handle negative situations well than people with poor EI are [26,27,28,29,30]. The overall finding also echoes trait emotional intelligence theory [19] and emotion theories [15,16], in which high trait emotional intelligence motivates flexible coping behaviors at school and protects students against problem behaviors. These results also support our hypothesis that adolescents with high EI are most capable of positive mental adjustment and have more positive social interactions than adolescents with low EI do [73,76], which can help them feel safe in their peer groups and avoid becoming the target of bullying.

### 4.1. Moderating Effects

The result shows that the strength of the link between emotional intelligence and school victimization decreases as the percentage of female participants increases. It suggests that sex is a moderator. One explanation is that the females’ EI is higher than the males’ EI is [62,63]. Another explanation could be that the prevalence of bullying victimization is lower among females than it is among males [64,65,66]. Friendships are valued highly among adolescents, particularly among females [82], and as a result, females may be less likely to report bullying incidents perpetrated by a friend for fear of social rejection [83], which weakens the correlation between EI and school victimization among females.

In this study, emotional intelligence measurement tools significantly moderate the link between emotional intelligence and school victimization. One possible explanation is that the EIS, EQI, TMMS, WLEIS, and SUEIT scales represent separate models. EIS and EQI belong to the self-reported mixed model, while TMMS, SUEIT, and WLEIS are in the self-report ability model. Variable models for testing emotional intelligence that emphasize different aspects might lead to the moderation effect.

However, bullying victimization types do not moderate the association between emotional intelligence and school bullying victimization, indicating that the link between EI and school bullying victimization remains stable regardless of traditional bullying victimization or cyberbullying victimization. Additionally, no significant differences emerged in the association between emotional intelligence and school bullying victimization across the bullying victimization measurement tools. Due to the fact that there must be at least three required entries to consider variability for the chi-square analysis to assess the presence of a moderator variable, the scales whose number was less than three were not categorized in the moderation analysis [71]. The bullying victimization measurement tools’ moderator analysis excluded 17 scales, while the EI measurement tools’ moderator analysis excluded 7 scales due to the mentioned reason. Thus, the results cannot precisely represent all 24 studies.

Further, the results of this study indicate that culture did not significantly moderate the link between emotional intelligence and bullying victimization, showing no support for the claim that the emotional intelligence and school bullying victimization link is stronger in Eastern countries than it is in Western countries [22,23,56].

In addition, the association between EI and bullying victimization did not differ across grade level, which is inconsistent with the group socialization development hypothesis [53]. The explanation could be that the group of university students was not included in the analysis because the scales whose number was less than three could not be categorized in the moderation analysis [71]. As a result, the outcome may be insignificant. This implies that reducing school bullying victimization through improving EI would be applicable for all age groups of children and youth students.

### 4.2. Limitations

This meta-analytic review has a few limitations that should be listed. First, the number of studies included in our meta-analysis was relatively small. Future meta-analyses could evaluate a broader range of moderators as more studies become available to provide more precise findings. Second, only three types of moderating factors were tested in this study: demographics, variable e-types, and measurement tools. We did not test whether sub-dimensions of EI could be used as a moderating variable because only a few studies reported correlations between different sub-dimensions of emotional intelligence and bullying victimization, and these sub-dimensions were not classified under the same model. Other potential moderating factors, such as bullying victimization informants, parenting styles, and ethnicity, should be investigated in future studies as more empirical studies demonstrating the value of the moderators above become available. Third, because most of the underlying studies were conducted at a single time, future longitudinal studies are needed to confirm the existing findings and identify the mechanisms by which an adequate level of EI is linked to a decrease in school bullying victimization among children and youth students.

## 5. Conclusions and Implications

To our knowledge, this is the first meta-analysis to examine the association between EI and school bullying victimization in children and youth students. Our meta-analysis found a small, negative, and significant association between emotional intelligence and bullying victimization, indicating that children and youth students with higher EI scores are less likely to be bullied at school. Sex and EI measurement tools were found to moderate the reported association in the moderators we examined. The findings showed that implementing emotional intelligence intervention programs in schools could reduce students’ risk of being bullied, and they would be more effective among male students.

This meta-analysis contributes to the existing research by showing that emotional intelligence is weakly negatively associated with school bullying victimization among children and youth students. The results suggest that high EI can decrease the likelihood of being bullied in school and online. The finding opens various new research areas and underscores the significance of EI as a crucial factor in addressing the emotional aspects of bullying victimization.

Policies and interventions aimed at reducing school and cyberbullying victimization should emphasize the importance of fostering students’ emotional intelligence, given that emotional intelligence has been proven to protect against bullying and cyberbullying victims. Increasing students’ EI levels may be a prospective aim of the intervention to prevent bullying. School professionals and practitioners can consider several effective school-based interventions and prevention programs to promote the students’ emotional intelligence. For example, the INTEMO program [84] based on Mayer and Salovey’s [40] ability theoretical model has shown positive effects on improving adolescents’ emotional intelligence levels and reducing aggressive behaviors [85,86]. Other school-based prevention programs that are in line with the constructs of EI, including The RULER Approach (“RULER”) designed for kindergarteners through to eighth graders, have been found effective in improving the students’ emotional abilities in recognizing, understanding, labeling, expressing, and regulating emotions [87,88]. The effectiveness of these intervention programs in reducing school bullying victimization would still need to be tested in China and other Asian countries. Furthermore, the association between EI and bullying victimization was larger among males than it was among females. The findings have sex-specific implications for schools, indicating that future EI interventions targeting to reduce school victimization may support male victims more effectively than they do female victims.

## Figures and Tables

**Table 1:** The summary of the 24 studies included in the meta-analysis.

Name (Year)	N	Age	Female%	Culture	Grade Level	Bullying Victimization Tools	EI Tools	Bullying Victimization Types	r
Alvarado, 2020 [49]	329	9.30	53	W	E	Bullying and school violence (AVE)	EMOCINE	T	−0.089
Baroncelli, 2014 [73]	529	12.60	53	W	S	Traditional bullying victimization scale (11 items); cyber victimization scale (10 items)	EIS	T, C	−0.100
BEKİR, 2021 [74]	272	NA	46	E	S	the Revised Cyber Bullying/Victimization Inventory-II	EQI	C	−0.130
Cañas, 2020 [56]	1318	13.8	53	W	S	PVS	TMMS	T	−0.050
Elipe, 2015 [28]	638	20.45	68	W	U	ECIP-Q	TMMS	C	−0.210
Extremera, 2018 [29]	1660	14.10	50	W	S	ECIP-Q	WLEIS	C	−0.130
Hsieh, 2019 [23]	6233	NA	50	E	E	PVS	EIS	T	−0.120
Kaynak, 2010 [75]	376	13.30	55	W	S	the Problem Behavior Frequency Scale (PBFS)	SREIT	T	−0.093
Kokkinos, 2012 [24]	206	NA	54	W	E	the Bullying and Victimization Scale	TEIQue	T	−0.260
León-del-Barco, 2020 [26]	822	10.58	46	W	E	School Coexistence Questionnaire (SCQ)	TMMS	T	−0.138
Lomas, 2012 [76]	68	13.85	54	W	S	PRQ	SUEIT	T	−0.211
Mavroveli, 2011 [21]	565	9.12	51	W	E	PVS, the five ‘Guess Who’ descriptions	SUEIT	T	−0.323
Martínez-Martínez, 2020 [55]	3451	15.73	47	W	S	Cybervictimization (26 items)	TMMS	C	−0.450
ÖNDER, 2017 [77]	545	12.48	52	W	S	Bullying Scale	EQI	T	−0.215
Peachey, 2017 [22]	235	NA	51	W	E	the Forms of Bullying Scale–Victim (FBS-V)	TEIQue	T	−0.090
Quintana-Orts, 2021a [34]	1929	14.65	52	W	S	EBIPQ, ECIP-Q	WLEIS	T, C	−0.055
Quintana-Orts, 2021b [78]	3520	14.37	52	W	S	EBIPQ, ECIP-Q	WLEIS	T, C	−0.101
Rey, 2018 [47]	1645	14.08	51	W	S	ECIP-Q	WLEIS	C	−0.074
Schokman, 2014 [35]	284	NA	21	W	S	PRQ	SUEIT	T	−0.224
Segura, 2020 [59]	1318	13.80	53	W	S	Cybervictimization (CYBVIC-R)	TMMS	C	−0.007
Tan, 2021 [57]	650	NA	52	E	S	Online Violence Fact Sheet 2017	WLEIS	C	−0.102
ŢEPORDEI, 2016 [79]	120	NA	54	W	S	Students’ Self-Report Questionnaire developed by Stevens (22 items)	EIS	T	−0.218
Vogel, 2006 [58]	235	NA	46	W	E	PRQ	EQI	T	−0.170
Yadav, 2018 [80]	490	NA	39	E	U	Cyber bullying victimization (9 items)	EIS	C	−0.100

Note. C = cyberbullying victimization; T = traditional bullying victimization; E = elementary school students; S = secondary school students; U = university students; EIS = Emotional Intelligence Scale; SUEIT = Swinburne University Emotional Intelligence Test; SREIT = The Self-Report Emotional intelligence Scale; TEIQue = Trait Emotional Intelligence Questionnaire; EQI = The Bar-On Emotional Quotient Inventory; TMMS = Trait Meta-Mood Scale; WLEIS = Wong Law Emotional Intelligence Scale; EMOCINE = EMOtion in CINEma scenes; ECIPQ = the European Cyberbullying Intervention Project Questionnaire; PRQ= peer relation questionnaire; PVS = Peer Victimization Scale; EBIPQ = The European Bullying Intervention Project Questionnaire.

**Table 2:** Tests of moderators of the bullying victimization links with EI.

	*Q*_between_	k	N	Mean r	SE	95% CI for r		*Q*_within_
						LL	UL	
EI tools	25.691 ***							
EIS		4	7372	−0.119	0.001	−0.141	−0.096	1.598
EQI		3	1052	−0.183	0.003	−0.241	−0.124	1.438
TMMS		5	7547	−0.177	0.048	−0.377	−0.039	329.454
WLEIS		5	9404	−0.092	0.001	−0.112	−0.072	6.015
SUEIT		3	917	−0.285	0.006	−0.344	−0.224	2.587
Bullying victimization tools	6.739							
ECIPQ		5	9392	−0.101	0.002	−0.145	−0.057	17.684
PRQ		3	587	−0.201	0.006	−0.278	−0.122	0.409
PVS		3	8116	−0.161	0.012	−0.273	−0.045	32.285
Bullying victimization types	0.062							
C		11	16,102	−0.131	0.018	−0.236	−0.022	464.044
T		16	17,314	−0.145	0.002	−0.178	−0.111	53.370
Grade level	0.645							
Elementary		7	8625	−0.170	0.005	−0.234	−0.104	28.588
Secondary		15	17,685	−0.144	0.015	−0.231	−0.055	465.686
Culture	1.238							
Eastern		4	7645	−0.118	0.001	−0.140	−0.095	0.395
Western		20	19,793	−0.161	0.012	−0.232	−0.088	484.803

Note. *** *p* < 0.001, EIS =Emotional Intelligence Scale; EQI = The Bar-On Emotional Quotient Inventory; TMMS = Trait Meta-Mood Scale; WLEIS = Wong Law Emotional Intelligence Scale; ECIPQ = The European Cyberbullying Intervention Project Questionnaire; PRQ = peer relation questionnaire; PVS = Peer Victimization Scale; C = Cyberbullying victimization; T = Traditional bullying victimization.

**Table 3:** Meta-regression analysis of female%.

Variable	Parameter	Estimate	SE	z-Value	95% CI for r	
					LL	UL
Female (%)	*β*_0_	0.008	0.001	6.281	0.005	0.010
	*β*_1_	−0.560	0.064	−8.725	−0.685	−0.434
*Q_Model_* (1, k = 24) = 502.541, *p* < 0.001						

## Data Availability

Not applicable.
